# Nomad Jellyfish *Rhopilema nomadica* Venom Induces Apoptotic Cell Death and Cell Cycle Arrest in Human Hepatocellular Carcinoma HepG2 Cells

**DOI:** 10.3390/molecules26175185

**Published:** 2021-08-26

**Authors:** Mohamed M. Tawfik, Nourhan Eissa, Fayez Althobaiti, Eman Fayad, Ali H. Abu Almaaty

**Affiliations:** 1 Department of Zoology, Faculty of Science, Port Said University, Port Said 42526, Egypt; n.essa@sci.psu.edu.eg (N.E.); ali_hussein@sci.psu.edu.eg (A.H.A.A.); 2 Department of Biotechnology, Faculty of Sciences, Taif University, P.O. Box 11099, Taif 21944, Saudi Arabia; faiz@tu.edu.sa

**Keywords:** *Rhopilema nomadica*, apoptosis, cell cycle arrest, HepG2

## Abstract

Jellyfish venom is a rich source of bioactive proteins and peptides with various biological activities including antioxidant, antimicrobial and antitumor effects. However, the anti-proliferative activity of the crude extract of *Rhopilema nomadica* jellyfish venom has not been examined yet. The present study aimed at the investigation of the in vitro effect of *R. nomadica* venom on liver cancer cells (HepG2), breast cancer cells (MDA-MB231), human normal fibroblast (HFB4), and human normal lung cells (WI-38) proliferation by using MTT assay. The apoptotic cell death in HepG2 cells was investigated using Annexin V-FITC/PI double staining-based flow cytometry analysis, western blot analysis, and DNA fragmentation assays. *R. nomadica* venom displayed significant dose-dependent cytotoxicity on HepG2 cells after 48 h of treatment with IC_50_ value of 50 μg/mL and higher toxicity (3:5-fold change) against MDA-MB231, HFB4, and WI-38 cells. *R. nomadica* venom showed a prominent increase of apoptosis as revealed by cell cycle arrest at G2/M phase, upregulation of p53, BAX, and caspase-3 proteins, and the down-regulation of anti-apoptotic Bcl-2 protein and DNA fragmentation. These findings suggest that *R. nomadica* venom induces apoptosis in hepatocellular carcinoma cells. To the best of the authors’ knowledge, this is the first scientific evidence demonstrating the induction of apoptosis and cell cycle arrest of *R. nomadica* jellyfish venom.

## 1. Introduction

Hepatocellular carcinoma (HCC) is the fourth most common cause of cancer-related mortality globally [1]. In Egypt, HCC is one of the prevalent malignancy cancer accounting for 70.48% of all liver tumors in both sexes, which may be attributed to the rising incidence rates of hepatitis C virus-related cirrhosis [2,3]. Chemotherapy and radiotherapy were determined to be the main rational therapeutic regimens for HCC [4,5]. However, the use of these routine treatments is assigned with potent toxic adverse effects in addition to the development of resistance of HCC cells to anticancer drugs [6,7]. In this perspective, there is an unmet need for novel anticancer agents that may have different mechanisms of action from current therapies, leading to higher selectivity for HCC cells.

Recently, jellyfish populations are rapidly increasing in various marine ecosystems worldwide, which may be associated with global warming, eutrophication, alien species invasions, and changes in salinity [8,9,10]. Jellyfish venom is primarily confined in specialized venom-containing capsules known as nematocysts that are found mainly in the tentacles. Nematocysts venom contains a mixture of bioactive proteins and peptides, which exhibits hemolytic, cardiotoxic, neurotoxic, musculotoxic, antioxidant, and cytolytic effects [11,12,13,14,15]. Jellyfish venom proteins are highly potent against a panel of human cancer cell lines which have attracted significant interests in tumor research [16,17].

Stimulating apoptotic pathways is the most significant non-surgical cancer treatment and HCC treatment in particular. Such strategies to trigger cancer cell death include upregulation of pro-apoptotic proteins and reduction of the expression of anti-apoptotic proteins [18,19,20]. Apoptosis is mainly associated with DNA fragmentation, chromatin compaction, nuclear fragmentation, and cell cycle arrest [21,22,23]. Interestingly, various jellyfish venoms have been reported to induce apoptosis and cell cycle arrest against diverse cancer cells [24,25]. Several studies have clearly indicated the link between the ability of jellyfish venom to produce oxidative stress and its induction of apoptosis in cancer cells [16,26,27]. ROS generation, lipid peroxidation induction, and mitochondria damage contribute to the cytotoxicity of the particular jellyfish venom, e.g., that of *Pelagia noctiluca* towards colon cancer cells and that of *Cassiopea andromeda* towards breast cancer cells, respectively [28,29].

*Rhopilema nomadica* jellyfish (nomad jellyfish) blooms off the eastern Mediterranean coasts of Italy, Turkey, Greece, Tunisia, and Egypt [30,31,32]. Nomad jellyfish was introduced in the Egyptian Mediterranean via Suez Canal in the latter half of the last century, which causes severe damage effects on the fishery industry and tourism [33,34]. Very few studies have explored the biological activities of nomad jellyfish. The present study is designed to assess the cytotoxic activity of *R. nomadica* venom on liver cancer cells (HepG2), breast cancer cells (MDA-MB231), human normal fibroblast (HFB4), and human normal lung cells (WI-38). The study also evaluates the extent of apoptosis in HepG2 cell death caused by *R. nomadica* venom.

## 2. Results

### 2.1. Electrophoretic Separation of the Protein Mixture of R. nomadica Venom by SDS-PAGE

**Figure 1:**
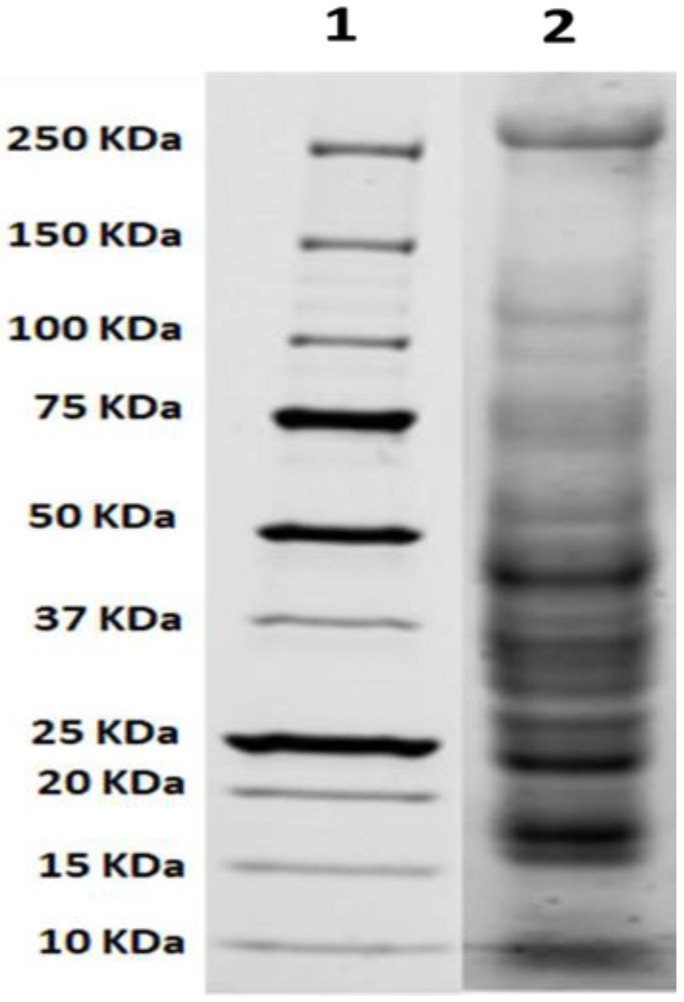
SDS-PAGE protein profile of *R. nomadica* venom. Lane 1: standard marker, Lane 2: protein extracts from the crude venom.

Quantitative analysis of proteins from *R. nomadica* venom was performed using the SDS-PAGE gel. The results revealed the presence of proteins ranging in mass from 16 to ~250 kDa. Major six protein bands with molecular masses of ~16, ~18, ~23, ~28, ~31, and ~48 kDa have dominated the profile (Figure 1).

### 2.2. R. nomadica Venom Suppresses the Growth of HepG2, MDA-MB231, HFB4 and WI-38 Cells

*R. nomadica* venom was evaluated for cytotoxic activity on the viability of the human hepatocellular carcinoma cell line (HepG2) using MTT assay. The venom has shown a significant cytotoxic effect on HepG2 cells in a concentration-dependent manner after 48 h of cell treatment. The IC_50_ value was approximately determined to be 50 μg/mL.

**Figure 2:**
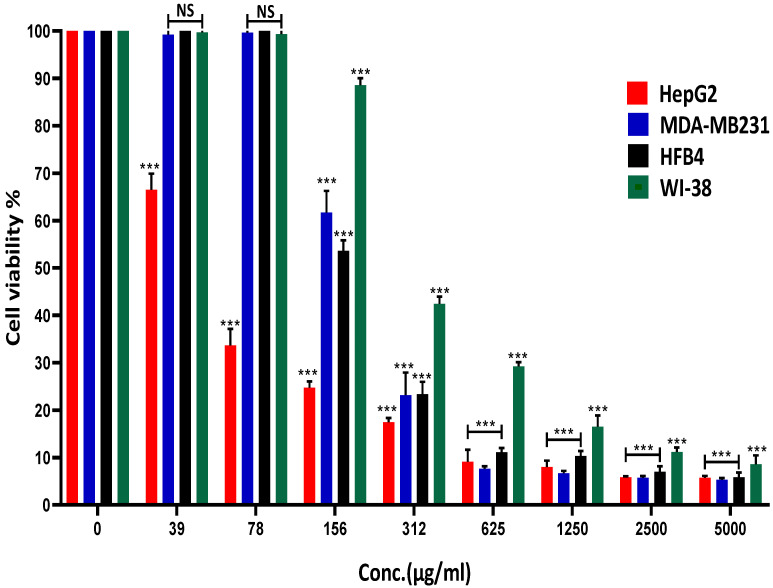
Effects of *R. nomadica* venom on cell proliferation of HepG2, MDA-MB231, HFB4, and WI-38 cell lines at different concentrations. The percentage of cell viability was measured using MTT assay after 48 h treatment. Non-treated cells were used as a control. Data are analyzed with one-way ANOVA followed by Tukey’s test. All values are represented as mean ± SEM. *** denotes significance difference (*p* < 0.001) vs. control, otherwise noted NS: non-significant (*p* > 0.05).

The toxicity testing of the venom was extended on extra cancer cell line MDA-MB231 (breast cancer) and two normal cell lines HFB4 (human fibroblast) and WI-38 (human lung cells). Results showed that MDA-MB231, WI-38, and HFB4 cells had an IC_50_ for the venom of 216, 250, and 168 µg/mL, respectively, 3:5-fold higher than HepG2 cells. Therefore, the crude extract of *R. nomadica* venom exhibited some preference against HepG2 cells rather than other cancer or normal cell lines (Figure 2).

### 2.3. R. nomadica Venom Induces G2/M Arrest on HepG2 Cells

**Figure 3:**
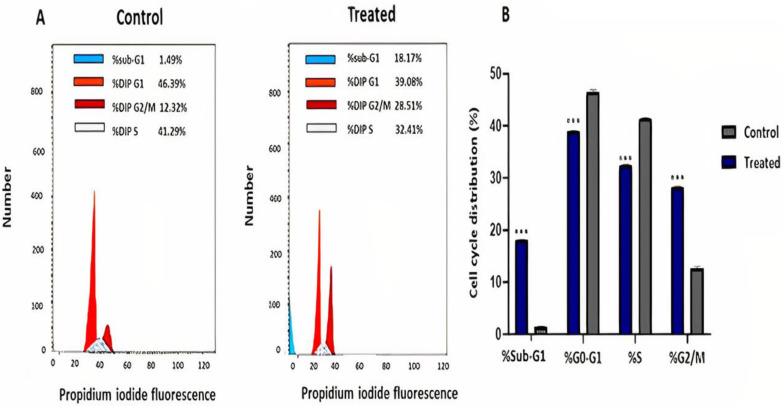
Cell cycle analysis of HepG2 cells treated with IC_50_ value of *R. nomadica* venom for 48 h by flow cytometry. (**A**) Representative profiles of cell cycle distribution in HepG2 cells after treatments. (**B**) The percentages of cell populations in sub-G1, G0–G1, S and G2/M phases. Data are analyzed with paired Student’s t-test. Values are represented as mean ± SEM. *** denotes significance difference (*p* < 0.001) vs. control.

Flow cytometric analysis of the cell cycle was performed to determine the cell cycle distribution of HepG2 cells treated with *R. nomadica* venom. The incubation of HepG2 cells with IC_50_ of *R. nomadica* venom for 48 h caused cell cycle arrest at the G2/M phase. The population of cells in G2/M and sub-G1 phases significantly increased from 12.28% and 1.49% in untreated cells to 28.51% and 18.17% in the treated cells with *R. nomadica* venom, respectively (*p* < 0.001). On the contrary, the treatment with *R. nomadica* venom resulted in a statistically significant decrease in the population of cells in G0–G1 and S phases (*p* < 0.001) (Figure 3).

### 2.4. R. nomadica Venom Induces Apoptosis in HepG2 Cells

**Figure 4:**
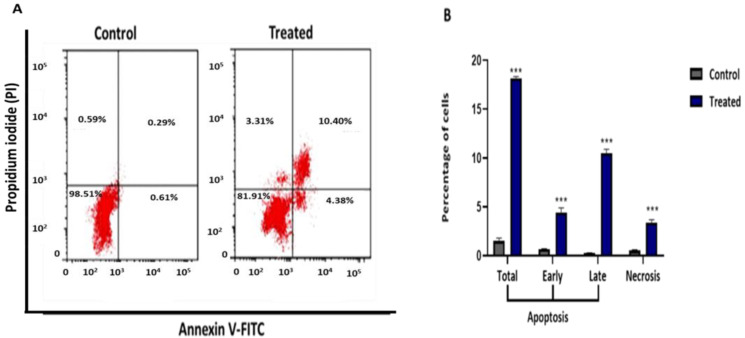
Apoptosis detection assay was performed using the Annexin V/PI double staining assay in HepG2 cells. HepG2 cells were treated with IC_50_ value of *R. nomadica* venom for 48 h, stained with Annexin V and PI, and analyzed on BD FACSCalibur flow cytometer. (**A**) Representative scatter plots of PI (*y*-axis) vs. Annexin V (*x*-axis). Lower left quadrants show viable cells (An −, PI −), whereas lower right quadrants represent the early apoptotic cells (An +, PI −). The upper left quadrants contain the necrotic cells (An −, PI +), while the upper right quadrants demonstrate the late apoptotic cells (An +, PI +). (**B**) Quantification graph of Annexin V/PI double staining assay obtained from BD FACSCalibur flow cytometer. Data are analyzed with paired Student’s t-test. Values are represented as mean ± SEM. *** denotes significance difference (*p* < 0.001) vs. control.

Annexin V-FITC/PI double staining-dependent on flow cytometry analysis showed that *R. nomadica* venom-induced apoptotic cell death in HepG2 cells (Figure 4). Populations of early and late apoptotic cells were significantly increased after 48 h treatment of IC_50_ of *R. nomadica* venom compared to untreated HepG2 cells (*p* < 0.001). Accordingly, the percentage of total apoptotic cells was significantly increased from 1.49% to 18.17% of treated cells.

### 2.5. R. nomadica Venom Induces Apoptosis in HepG2 Cells through the Regulation of the Expression of Apoptosis-Related Proteins

**Figure 5:**
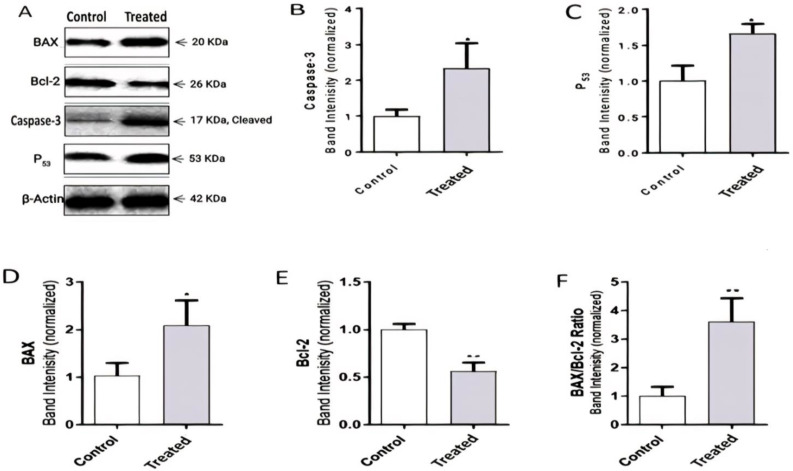
Western blot analysis. (**A**) shows western blot results about the expression level of apoptosis-related proteins BAX, Bcl-2, caspase-3, and p53 in HepG2 cells after being treated for 48 h with IC_50_ concentration of *R. nomadica* venom. From (**B**–**F**) represent the statistical graphs of the density ratios of the proteins calculated by ImageLab. Protein levels were normalized to β-Actin. Data are analyzed with paired Student’s t-test Values are represented as mean ± SEM. * denotes significance difference (*p* < 0.05) vs. control. ** denotes significance (*p* < 0.01) vs. control.

To understand the molecular mechanisms underlying *R. nomadica* venom-induced apoptosis, western blotting was performed to investigate the expression levels of apoptosis-related proteins. The expression of anti-apoptotic Bcl-2 protein was remarkably decreased in *R. nomadica* venom-treated HepG2 cells after 48 h treatment with IC_50_ concentration. Whereas apoptosis-inducing proteins BAX, caspase-3, and p53 were increased in the treated cells. Upon treatment of HepG2 cells with *R. nomadica* venom, a significant increase in Bax/Bcl-2 ratio compared to untreated cells (*p* < 0.01) (Figure 5).

### 2.6. R. nomadica Venom Induces DNA Damage

**Figure 6:**
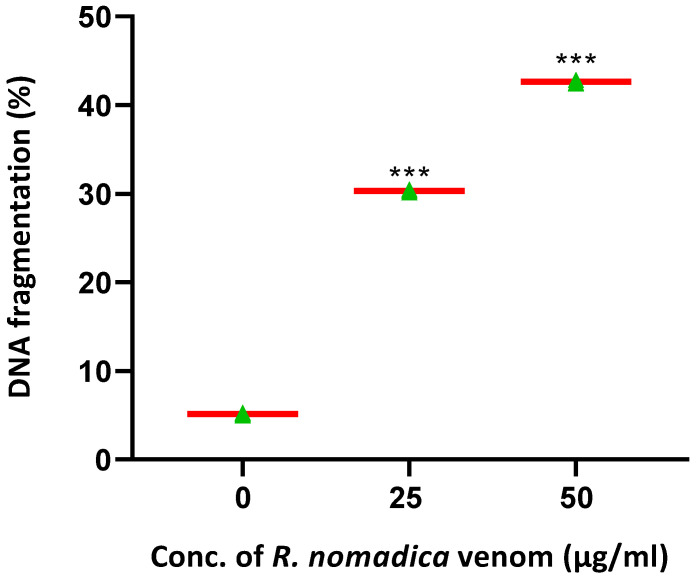
Quantitative estimation of DNA fragmentation by diphenylamine (DPA) assay in treated HepG2 cells with two different concentrations of *R. nomadica* venom (25–50 μg/mL) and untreated cells. Data are analyzed with one-way ANOVA followed by Tukey’s test. Values are represented as mean ± SEM. *** denotes significance difference (*p* < 0.001) vs. control.

The DPA assay measured the relative quantity of DNA fragments in the treated HepG2 cells with *R. nomadica* venom (Figure 6). Compared to the untreated cells (5.27% ± 0.29%), *R. nomadica* venom at concentrations of 25 and 50 μg/mL caused a marked significant elevation in DNA fragmentation percentage (30.47% ± 1.66%) and (42.85% ± 2.33%), respectively.

**Figure 7:**
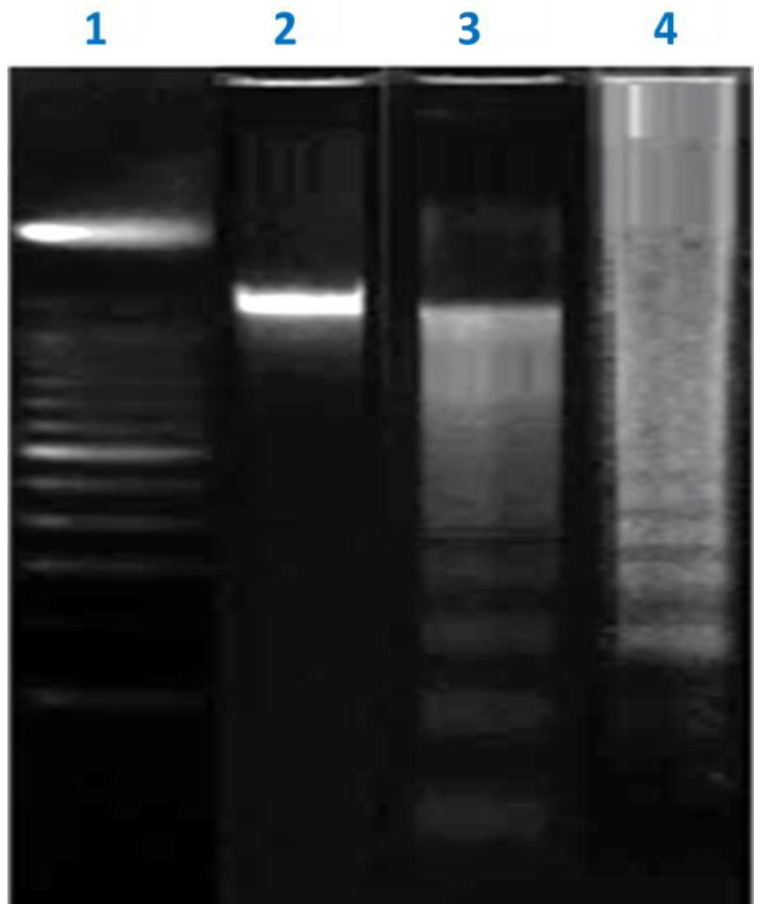
Electrophoretic pattern of DNA fragments isolated from HepG2 cells on 8% agarose gel electrophoresis. Lane 1: DNA marker, 100-bp. Lane 2: the intact DNA of the untreated HepG2 cells. Lane 3 and 4: the fragmented DNA of HepG2 cells treated with 25 and 50 μg/mL of *R. nomadica* venom, respectively.

Agarose gel electrophoresis of DNA from HepG2 cells treated with *R. nomadica* venom showed a ladder-like pattern of DNA fragments in a concentration-dependent manner relative to untreated cells (Figure 7). The gel pattern of the DNA samples isolated from untreated control of HepG2 cells showed clear bands of intact DNA, while *R. nomadica* venom-induced an increase in DNA smearing level with increasing dose from 25 to 50 μg/mL.

### 2.7. R. nomadica Venom Induces Less Hemolytic Activity against Human Erythrocytes

**Figure 8:**
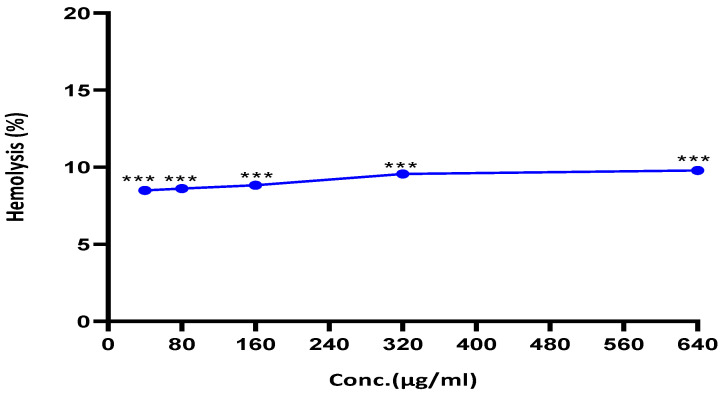
The hemolytic activity of *R. nomadica* venom against human RBC_s_ incubated with serial concentrations of the crude venom for 1 h at room temperature. PBS and 10% triton 100× were used as negative and positive controls, respectively. The absorbance of the supernatant was measured at 570 nm. Data are analyzed with one-way ANOVA followed by Tukey’s test. Values are represented as mean ± SEM. *** denotes significance difference (*p* < 0.001) vs. control.

The hemolysis assay is carried out to investigate whether the cytotoxic activity is related to direct damage to the cell membrane. *R. nomadica* venom exhibited weak hemolytic activity under 10% at tested concentrations from 40 to 640 µg/mL (Figure 8).

## 3. Discussion

Jellyfish venoms have long attracted the interest of researchers to find and develop novel anticancer agents. Jellyfish venoms either or isolated peptides have anti-proliferative effects on various cancer cell lines such as brain, colorectal, breast, lung, and liver cancer cells [26,28,35,36,37]. Although there are many swarms of *R. nomadica* jellyfish in the summertime of the Egyptian Mediterranean coasts, much less attention has been paid to their biological activities. The present study showed that crude venom of *R. nomadica* jellyfish inhibited the growth of HepG2 cells in a dose-dependent manner (IC_50_ value of 50 μg/mL). Less or no hemolytic activity has been detected for *R. nomadica* venom against human erythrocytes up to 10-fold greater than the IC_50_ concentration. To our knowledge, the anticancer activity of this venom is being reported herein for the first time.

Similar inhibitory effects against HepG2 cells have been reported for other jellyfish species such as *Nemopilema nomurai*, *Cyanea lamarckii,* and *Acromitus flagellates* venoms [35,37,38,39,40]. Interestingly, both *Acromitus flagellates* venom and *Cyanea lamarckii* extract share three to six protein bands with *R. nomadica* venom in their electrophoretic patterns [35,39]. A pore-forming toxin, CcTX-1 (31.17 KDa), is an isolated proteinaceous cytotoxin from *Cyanea capillata* jellyfish venom that exhibited potent inhibitory activity against HepG2 cells [41,42]. The other two cytotoxic proteins, known as CfTX-A (~40 kDa) and CfTX-B (~42 kDa), were partially purified from *Chironex fleckeri* venom [43]. Similar protein bands with a similar molecular weight of CcTX-1, CfTX-A, and CfTX-B have been observed in the protein profile of *R. nomadica* venom. These latter findings may interpret the cytotoxic properties of *R. nomadica* venom against HepG2 Cells.

In the current study, the DNA fragmentation assays revealed that treated HepG2 cells with IC_50_ value of *R. nomadica* venom showed a highly significant DNA fragmentation in comparison to non-treated cells. Similar DNA damages have been observed for *Nemopilema nomurai*, *Chiropsalmus quadrumanus* crude venoms and *Chrysaora Quinquecirrha* venom peptide against a variety of cancer cells particularly HCC cells [37,44,45].

Besides the DNA damage, cell cycle arrest is also a key event in apoptosis. Our results showed the induction of cell cycle arrest at the G2/M phase after the treatment of HepG2 cells for 48 h with IC_50_ value of *R. nomadica* venom and elevation in the sub-G1 population. These data are in accordance with the previous study that exhibited the increase of sub-G1 population in HepG2 cells treated with *Nemopilema nomurai* venom [37]. DNA fragmentation and cell cycle arrest results were denoting the strong apoptotic effect of *R. nomadica* venom.

Annexin V and PI double staining assay elucidated the efficacy of *R. nomadica* venom on triggering apoptosis in HepG2 cells treated with IC_50_ by raising the percentage of early and late apoptotic cells compared to untreated cells. Similar reports presented the venom potency of different jellyfish species such as *Stomolophus nomurai*, *Chrysaora helvola*, *Chiropsalmus quadrigatus*, *Chrysaora Quinquecirrha,* and *Cassiopea andromeda* in inducing the apoptotic cell death in various cancer cells via the formation of apoptotic bodies [36,44,45,46,47].

Apoptosis regulation is an impressive target for HCC treatment. Apoptosis is a significant mode of programmed cell death characterized by distinct hallmarks and controlled by essential extrinsic and intrinsic regulatory proteins [48,49]. The intrinsic pathway is mediated by pro-apoptotic proteins such as BAX/BAK proteins and anti-apoptotic proteins involved Bcl-2 and Bcl-xL proteins [50,51]. Terminally, apoptosis is executed by caspase-activated cascade involved the effector caspases such as caspase-3, 6, 7, and 10, which induce the activation of cytoplasmic endonuclease (CAD), which in turn causes chromatin condensation, DNA fragmentation, the formation of cytoplasmic blebs, and apoptotic bodies [52,53,54]. Furthermore, p53 is a tumor suppressor protein. Under Extra and intracellular stress signals, it plays a pivotal role in suppressing anti-apoptotic Bcl-2 family proteins. Moreover, p53 can directly interact with BAX and promotes the release of cytochrome c via mitochondrial outer membrane permeabilization (MOMP), which upregulates the tumor cells apoptosis [51,55].

Likewise, *R. nomadica* venom induces a potent intrinsically apoptotic effect on HepG2 cells by increasing p53 expression level, which in turn upregulates BAX (pro-apoptotic protein) and downregulates Bcl-2 (anti-apoptotic protein). We also demonstrate the intrinsic apoptotic pathway induced by *R. nomadica* venom in HepG2 cells through the elevation of BAX/Bcl-2 ratio leading to triggering of caspase-3 signaling protein, which causes cells destruction and ends up to apoptosis. Following the present results, previous studies have demonstrated the activity of other jellyfish venoms on modulating the apoptotic cell death in cancer cells via executioner and regulatory proteins [36,40,44,45,46]. Similarly, some apoptosis-inducing activities of other jellyfish venoms were attributed to their ability to produce high levels of ROS in different cancer cells, which may point out the potential ROS mediated cytotoxicity of *R. nomadica* venom [27,28,29].

Most of the jellyfish venoms which exhibited potent anticancer activities have strong hemolytic activities such as *Palythoa caribaeorum*, *Nemopilema nomurai,* and *Cassiopea xamachana* [26,56]. Even *Nemopilema nomurai* (schyphozoa) showed similar selective anticancer activities of *R. nomadica* venom against HepG2 cells rather than other cancer or normal cell lines [37]. However, erythrocytes were more susceptible to *N. nomurai* as >50% of mammalian RBCs underwent hemolysis at 100–200 µg/mL. In comparison with other jellyfish venoms and scyphozoans, *R. nomadica* showed less (weaker) hemolytic activities against RBCs. Furthermore, severe cytotoxicity on cardiac and skeletal cell lines has been reported for *N. nomurai* [12].

## 4. Materials and Methods

### 4.1. Jellyfish Collection

*R. nomadica* jellyfish specimens were captured from the eastern Egyptian Mediterranean of Port Said coast during the blooming summer months in 2019. Only tentacles were collected in tanks full of fresh seawater and transported immediately to a laboratory for further preparations.

### 4.2. Nematocysts Venom Extraction and Preparation

Nematocysts were isolated from the excised tentacles as described with slight modification [57]. Briefly, dissected tentacles were submerged in cold seawater at the mass: volume ratio of 1:3 to allow autolysis of the tissues at 4 °C with gentle swirling for 30 min every two hours. This process was repeated for 3 days. The nematocysts suspension was filtered through a plankton net to remove tissue debris and centrifuged at 10,000 rpm for 30 min at 4 °C. Then, the resultant supernatant was collected, lyophilized, and stored at −20 °C.

The crude venom was extracted from freeze-dried nematocysts with a minor modification. In which, 100 mg nematocyst powder was resuspended in 1 mL of phosphate-buffered saline (PBS, pH 7.4, 4 °C) and centrifuged at 15,000 rpm for 15 min at 4 °C [58]. The supernatant was separated and used as the extracted *Rhopilema nomadica* venom stock solution for the present study. The stock solution was aliquoted (to avoid freezing and rethawing) and stored at −20 °C. The protein concentration of the venom was estimated by the Bradford method, and the venom was used based on its protein concentration [59]. In terms of quality control, SDS-PAGE (Sodium dodecyl sulfate-polyacrylamide gel electrophoresis) (Bio-Rad Laboratories, Inc., Hercules, CA, USA) was used regularly to assess the banding pattern of venom proteins. Moreover, IC_50_ (concentration of the venom required for 50% inhibition of cell growth) against cell lines has been monitored to check the effectiveness and stability of the venom.

### 4.3. SDS-Polyacrylamide Gel Electrophoresis

*R. nomadica* crude venom proteins were separated based on their molecular weights by using SDS-PAGE. This technique was carried out using 12% polyacrylamide gel performed by TGX Stain-Free™ FastCast™ Acrylamide Kit (Bio-Rad Laboratories, Inc., Hercules, CA, USA). Briefly, the sample was boiled at 95 °C for 5 min with the sample loading buffer (4% SDS, 10% 2-mercaptoethanol, 20% glycerol, 0.004% bromophenol blue and 0.125 M Tris HCl, PH 6.8). The protein sample was electrophoresed for 60 min at 150 V, using Tris-glycine running buffer. Protein bands were visualized using stain-free technology and Chemi Doc imager (Bio-Rad Laboratories, Inc., Hercules, CA, USA) [60].

### 4.4. Cell Culture

HepG2 (human hepatocellular carcinoma), MDA-MB-231 (human breast adenocarcinoma), HFB4 (human normal fibroblast), and WI-38 (human normal lung) cell lines used in the present study were purchased from Holding company for biological products and vaccines (VACSERA, Giza, Egypt). Cells were maintained as monolayer in culture in complete medium (RPMI-1640 medium supplemented with 2 mM L-glutamine, 10% heat-inactivated FCS, 100 mg/mL streptomycin, and 100 U/mL penicillin) and were incubated at 37 °C with 5% CO2 in a humidified atmosphere.

### 4.5. Cell Viability by MTT Assay

To evaluate the potential cytotoxic effect of the *R. nomadica* venom on cell viability, an MTT (3-(4, 5-methylthiazol-2-yl)-2, 5-diphenyl-tetrazolium bromide) reduction assay was performed [61,62]. HepG2, MDA-MB-231, HFB4, and WI-38 cells (VACSERA, Giza, Egypt) were seeded in a 96-well plate at a concentration of 1.0 × 10^4^ cells/well and incubated at 37 °C for 24 h to settle down. The cells were treated for another 48 h with different concentrations ranging from 0 to 5000 µg/mL of *R. nomadica* venom. 20 μL of MTT solution was added to each well and incubated for an additional 3 h at 37 °C. After the supernatant was removed, dimethyl sulfoxide (200 µL DMSO) was added to each well to resuspend the formazan crystals. Cell viability was determined by measuring optical density at 540 nm using Bio-Tek ELISA multi-well plate reader (Bio-Tek Instruments Inc., Burlington, VT, USA). The cytotoxic effect of the crude venom was determined by comparing the optical density of the treated cells and the untreated cells. Phosphate buffered saline (PBS) was used as a negative control. The IC_50_ value was calculated from the dose-response curve.

### 4.6. Cell Cycle Analysis

Flow cytometry assessed the percentage of cellular DNA content was assessed by flow cytometry [37]. HepG2 cells were seeded in a 6-well plate at a density of 1.0 × 10^4^ cells/well and incubated at 37 °C for one day before the experiment. The cells were treated with IC_50_ concentration of *R. nomadica* venom for 48 h, harvested, and fixed with 70% cold ethanol at –20 °C overnight. Fresh PBS washed the fixed cells, then stained with PI (50 μg/mL) in the presence of RNase A (10 μg/mL) and incubated for 30 min in the dark. The cellular DNA content was analyzed using a flow cytometer (FACSCalibur, Becton Dickinson, Franklin Lakes, NJ, USA).

### 4.7. Annexin V-FITC/PI Double Staining Assay

HepG2 cells were treated with IC_50_ concentration of *R. nomadica* venom for 48 h. Both adherent and suspended cells were collected, centrifuged (1000 rpm for 5 min), washed with PBS and resuspended with binding buffer (1×). After that, the cells were stained with 4 µL Annexin V-FITC and 2 µL propidium iodide (PI) (Annexin V-FITC Apoptosis Detection Kit) (Abcam, Cambridge, UK) and incubated for 15 min at 37 °C in the dark. The total apoptotic and necrotic cells were measured by BD FACSCalibur flow cytometer [46].

### 4.8. Western Blot Analysis

HepG2 cells were treated with IC_50_ concentration of *R. nomadica* venom for 48 h, and the cell lysate was prepared in cold lysis buffer [100 mM NaCl, 10 mM Tris, 25 mM EDTA, 25 mM EGTA, 1% Triton X-100, 1% NP-40 (pH 7.4)], with 1:300 protease inhibitor cocktail (Sigma-Aldrich, St. Louis, MO, USA) and Phosphatase inhibitor cocktail Tablet (Roche Diagnostics GmbH, Mannheim, Germany). Total protein concentration was determined using the Bradford method before proceeding to the western blotting. Equal amounts (20 µg) of protein samples were loaded into 12% SDS-polyacrylamide gel and separated by Cleaver electrophoresis unit (Cleaver Scientific Ltd., Rugby, UK). They were transferred onto polyvinylidene fluoride (PVDF) membranes (Bio-Rad Laboratories, Inc., Hercules, CA, USA) for 30 min using a Semi-dry Electroblotter (BioRad) at 2.5 A and 25 V for 30 min. The membrane was blocked with 5% nonfat dry milk in TBS-T for two hours at 37 °C. The membrane was incubated overnight at 4 °C with primary antibodies against BAX (1:1000, Cell Signaling Technology, Inc., Danvers, MA, USA), Bcl-2 (1:1500, Cell Signaling Technology), cleaved caspase-3 (1:750, Cell Signaling Technology), p53 (1:1000, Abcam, Cambridge, UK), and β-actin (1:5000, Sigma-Aldrich, St. Louis, MO, USA). Then, the membrane was washed three times with TBS-T and followed by incubation with the corresponding horseradish peroxidase-linked secondary antibodies (1:1000, Dako Ltd., High Wycombe, UK) for another hour at room temperature. β-actin was used as an internal reference protein. Finally, specific protein bands were visualized by a chemiluminescent detecting kit (Perkin Elmer, Waltham, MA, USA). The blot image was captured using a CCD camera-based imager (Chemi Doc imager, BioRad). The bands' intensities were then measured by densitometry using ImageLab software (Bio-Rad Laboratories, Inc., Hercules, CA, USA) [63,64].

### 4.9. DNA Damage Evaluation

The apoptotic effect induced in HepG2 cells was evaluated by two different techniques that included diphenylamine (DPA) assay and DNA laddering assay.

#### 4.9.1. DNA Fragmentation Percentage by Diphenylamine (DPA) Assay

DPA assay is a colorimetrical quantitative method used for assessing the DNA fragmentation caused by the treatment of HepG2 cells by *R. nomadica* venom with the concentration of 25, 50 µg/mL for 48 h upon utilizing diphenylamine (DPA) reagent, which binds to deoxyribose [65]. The optical density was measured at 600 nm in the S (fragmented DNA) and the P (intact DNA) fractions. The percentage of fragmented DNA was calculated using the formula:

DNA fragmentation (%) = [S/(S + P)] × 100(1)

#### 4.9.2. DNA Laddering Assay

Genomic DNA was extracted from HepG2 cells after the treatment with three different concentrations of *R. nomadica* venom (25, 50 µg/mL) for 48 h. Electrophoresis was performed in 8% agarose gel with ethidium bromide staining. Following this, the DNA in gel was visualized under UV and photographed by a digital camera (Canon U.S.A., Inc., Lake Success, NY, USA) [66].

### 4.10. Hemolysis Assay

Hemolytic activity of *R. nomadica* venom was performed on erythrocytes of humans with some modifications [56]. A blood sample was taken from a healthy adult volunteer after informed consent was obtained. The blood sample was immediately combined with anti-clot material (EDTA) and centrifuged at 5000 rpm for 5 min. Briefly, the erythrocytes suspension in sterile phosphate buffer saline (PBS) was received serial concentrations of the crude venom. After one hour of incubation at room temperature, the cells were centrifuged, and the supernatant was used to measure the absorbance of the liberated hemoglobin at 570 nm. The positive control (100% hemolysis) and the negative control (0% hemolysis) were Triton X-100 and sterile phosphate buffer saline (PBS), respectively. The following equation measured the hemolysis percentage for each sample:

Hemolysis (%) = [(AS − A)/(AP.C − AN.C] × 100(2)

where AS is the mean of absorbance of sample, AN.C is the mean of absorbance of negative control, and AP.C is the mean of absorbance of positive control.

### 4.11. Statistical Analysis

All experiments are achieved in triplicate. The values are shown as mean value ± SE and data were analyzed using paired Student’s t-test and one-way ANOVA followed by Tukey’s test. Statistical significance was assessed by *p* < 0.05.

## 5. Conclusions

In conclusion, our findings highlighted the anti-proliferative effect of *R. nomadica* venom and clearly showed that the venom extract could induce apoptosis in vitro. Therefore, these data might provide a starting point for the research on using *R. nomadica* venom in the treatment of cancer and hepatocellular carcinoma in particular. However, future investigations may be conducted to investigate the anticancer activities of *R. nomadica* venom in vivo and to characterize and identify the bioactive peptides that exert anticancer activity. Further, future experiments will be planned to investigate cytoprotective properties of *R. nomadica* venom on H_2_O_2_-treated cells in vitro and in vivo models. Moreover, protein and gene expressions of the antioxidative enzymes and oxidative biomarkers for cell membrane lipid peroxidation will be analyzed upon treatment with *R. nomadica* venom. Such experimental work may reveal in detail the redox activity of *R. nomadica* venom.

## Data Availability

The data presented in this study are available on request from the corresponding author.

## Sample Availability

Samples of the compounds are available from the authors.
